# Performance of Four Transport and Storage Systems for Molecular Detection of Multidrug-Resistant Tuberculosis

**DOI:** 10.1371/journal.pone.0139382

**Published:** 2015-10-02

**Authors:** Marie Sylvianne Rabodoarivelo, Bélen Imperiale, Rina andrianiavomikotroka, Angela Brandao, Parveen Kumar, Sarman Singh, Lucilaine Ferrazoli, Nora Morcillo, Voahangy Rasolofo, Juan Carlos Palomino, Peter Vandamme, Anandi Martin

**Affiliations:** 1 Institut Pasteur de Madagascar, Antananarivo, Madagascar; 2 Hospital Dr. Cetrángolo, Buenos Aires, Argentina; 3 Instituto Adolfo Lutz Núcleo de Tuberculose e Micobacteriosis Sao Paulo, Sao Paulo, Brazil; 4 Division of Clinical Microbiology & Molecular medicine, All India Institute of Medical Sciences, New Delhi, India; 5 Laboratory of Microbiology, Department of Biochemistry and Microbiology, Ghent University, Gent, Belgium; 6 Fundação Oswaldo Cruz, IOC, Sao Paulo, Brazil; University of Delhi, INDIA

## Abstract

**Background:**

Detection of drug-resistant tuberculosis is essential for the control of the disease but it is often hampered by the limitation of transport and storage of samples from remote locations to the reference laboratory. We performed a retrospective field study to evaluate the performance of four supports enabling the transport and storage of samples to be used for molecular detection of drug resistance using the GenoType MTBDR*plus*.

**Methods:**

Two hundred *Mycobacterium tuberculosis* strains were selected and spotted on slides, FTA cards, GenoCards, and in ethanol. GenoType MTBDR*plus* was subsequently performed with the DNA extracted from these supports. Sensitivity and specificity were calculated and compared to the results obtained by drug susceptibility testing.

**Results:**

For all supports, the overall sensitivity and specificity for detection of resistance to RIF was between 95% and 100%, and for INH between 95% and 98%.

**Conclusion:**

The four transport and storage supports showed a good sensitivity and specificity for the detection of resistance to RIF and INH in *M*. *tuberculosis* strains using the GenoType MTBDR*plus*. These supports can be maintained at room temperature and could represent an important alternative cost-effective method useful for rapid molecular detection of drug-resistant TB in low-resource settings.

## Introduction

Tuberculosis (TB), and the emergence of multidrug-resistant (MDR)- and extensively drug-resistant (XDR)-TB, remains a global health concern, which poses an important challenge for TB control programs. It is estimated that two billion people are currently infected with *Mycobacterium tuberculosis* (MTB) with 10% of this population at risk of developing the active form of the disease during their lifetime. According to the last report of the World Health Organization (WHO), there were 9.0 million new TB cases in 2013 and 1.5 million TB deaths [1]. TB control efforts are based on the diagnosis of cases followed by adequate treatment.

Usually, in less-developed settings, cultures are not performed to detect possible drug resistance. Consequently, initial treatment of the disease is performed before the results of drug susceptibility testing (DST) are available or treatment gets delayed. Yet, rapid detection of drug resistance is critical for achieving favorable clinical outcomes and preventing the continued transmission of disease. The obstacles are that DST based on culture takes 3–8 weeks before obtaining the results, as MTB grows very slowly [2]. DST by culture is a long process and testing also requires a well-equipped biosafety level 3 laboratory, with well-prepared personnel and dedicated equipment. Moreover, in low-income countries, performing this type of tests is often restricted by constraints of both sputum storage and safe transport from peripheral health centers to central laboratories.

Over the last decade, the contribution of molecular methods for the diagnosis of TB has increased significantly. Molecular assays that can rapidly amplify DNA have been shown to be a promising alternative especially for developing countries [3]. One of these molecular methods is the GenoType MTBDR*plus* (Hain Lifesciences GmBH, Nehren, Germany). The method combines multiplex polymerase chain reaction (PCR) and DNA line-probe assay to identify genetic mutations conferring rifampicin (RIF) and isoniazid (INH) resistance and can be used on both cultures and directly on specimens [4]. Microscopy glass slides from routine smear examination are normally not infectious and susceptible to be transported to other laboratories in other locations without the need for preservation or costly cold chain. Consequently, they could represent an ideal material for recovering DNA to be used in downstream molecular tests. Previous studies have shown their utility for extracting DNA from mycobacteria from several sources [5–9]. Other systems based on filters such as the FTA card (Whatman International Ltd, UK) and the GenoCard (Hain Lifescience, Nehren, Germany) have also been tested to send samples to a reference laboratory by mail from remote locations [10–11]. They could represent a useful tool for collection and transport of clinical specimens to reference laboratories for a quick detection of drug-resistant TB. The amplification of the DNA is made by detaching a small disc from the seeded area of the card, and by directly transferring it to the amplification mix for PCR. To overcome difficulties of transporting and storing samples using simple ways for downstream DNA-based testing, we performed the first multicenter field evaluation study comparing DNA extracted from Ziehl-Neelsen (ZN) stained smear slides, from strains spotted on two commercial card-systems: FTA card and GenoCard, and from MTB strains kept in ethanol, for the subsequent identification and genotypic detection of drug-resistant TB using the GenoType MTBDR*plus*.

## Methods

### Study sites

This study was conducted at four participating sites: Mycobacteriology Unit, Institute Pasteur of Madagascar (IPM), Antananarivo, Madagascar; Division of Clinical Microbiology and Molecular Medicine, All India Institute of Medical Sciences (AIIMS), New Delhi, India; Mycobacteria Reference Laboratory of Buenos Aires Tuberculosis Control Program Hospital Dr. Cetrángolo, Buenos Aires, Argentina; Núcleo de Tuberculose e Micobacteriosis, Instituto Adolfo Lutz, Sao Paulo, Brazil.

### Study design

The study was divided in 3 phases. **Phase 1** involved 2 parts. In part 1, four non-tuberculous mycobacteria (NTM) strains *(M*. *chelonae*, *M*. *avium*, *M*. *abscessus and M*. *kansasii)* were chosen for the standardization of the DNA extraction technique from the 4 different storage and transport systems: (1) ZN stained smear slides, (2) FTA cards (3) GenoCards, and (4) from ethanol. The second part involved the evaluation of six MTB strains from which, three were MDR and three susceptible, to validate the protocol previously standardized in part 1. In **phase 2**, the biosafety related to the viability of the bacilli in the stored samples on the 4 different storage systems was evaluated using the reference strain MTB H37Rv. **Phase 3** was the field evaluation study at the four participating sites using the GenoType MTBDR*plu*s to detect drug resistance (RIF and INH) from 50 DNA extracted from the 4 different storage and transport systems. In this study we use the term of “strains” through all the text which means culture isolates.

### Phase 1: Standardization of DNA extraction from different storage and transport system

#### Part 1: NTM DNA extraction

**Mycobacterial cell suspensions:** Four reference NTM strains: *M*. *chelonae ATCC 14472*, *M*. *avium CCUG 27851*, *M*. *abscessus CCUG 41449* and *M*. *kansasii CCUG 32245* were chosen for the standardization of DNA extraction from the different storage systems. Bacterial suspensions were prepared by resuspending 1 μl loopful of cells scraped from Löwenstein-Jensen (LJ) medium in 0.5 ml sterile distilled water. Cell suspensions were vortexed and adjusted to the scale of McFarland 1 (10^7^–10^8^ CFU/ml). One hundred microliters of the bacterial suspension was spotted on one microscopy slide, 60 μl on one GenoCard (1 sample area, 1 cm diameter), 100 μl on one FTA card (1 sample area, 125 μl size, 2.5 cm diameter) and 100 μl in 500 μl of absolute ethanol and stored at room temperature until use. For the GenoCard due to the small area available for spotting the sample, a total volume of 60 μl of bacterial suspension was added by spotting 20 μl in three times, letting dry between each time. Both filter cards were air dried at room temperature for 15 min in the biosafety cabinet before being stored in sealed plastic bags. For the negative control, one drop of sterile distilled water was spotted in each system.

**DNA extraction from ZN stained smear slides:** The Chelex method was used according to Dubois et al. (2011) with small modifications [5]. The oil immersion from the slide was first removed with Xylene. Twenty five microliter of sterile distilled water was then added to the slide and the smear scraped off using a sterile scalpel. The suspension was transferred to a 1.5 ml microtube containing 75 μl of 10% Chelex-100. The microtube was then incubated at 95°C for 30 min then thoroughly mixed and centrifuged at 13000 rpm for 10 min. The supernatant containing the DNA was transferred to a new microtube and stored at -20°C until further use.

**DNA extraction from GenoCard:** The DNA extraction from GenoCard was performed according to Miotto et al. (2008) with small modifications [11]. Briefly, 60 μl of the bacterial suspension McFarland 1 were spotted directly on the GenoCard sample area as described above. Using a special punch (Hain Lifescience, Germany), 8 small discs were punched from the sample spotted area and placed in a microtube. To ensure that there is no cross-contamination between punched discs from different strains, the cutting end of the punch was rinsed with 70% ethanol each time. Sixty microliter of 10% Chelex-100 was added in the microtube, mixed and incubated at 95°C for 30 min. The microtube was then centrifuged at 13000 rpm for 10 min, and the supernatant transferred to a new microtube and stored at -20°C until use.

**DNA extraction from FTA card:** DNA extraction from FTA card was performed according to Guio et *al*. (2006) with small modifications [10]. Briefly, 100 μl of the bacterial suspension McFarland 1 were added on the FTA card sample area as described above. With a Harris microPunch the card was perforated and discs were transferred to a microtube. 1 disc was initially punched and tested. The number of discs was then increased to 4 after PCR amplification result from disc 1. Before each punching, we rinsed the end of the punch (Whatman International Ltd, UK) with 70% alcohol to avoid cross contamination. Discs were rinsed 3 times with 200 μl of Whatman FTA purification reagent (GE Healthcare Life Sciences) to remove PCR inhibitors and other potential contaminants and to ensure the quality of DNA, and finally rinsed twice with 200 μl of TE buffer (10 mM TRIS, 1 mM EDTA, pH 8). TE buffer was discarded by pipetting after a short spin at 3000×g and finally 75 μl 10% Chelex-100 was added to the microtube, mixed and incubated at 95°C for 30 min. After centrifugation at 13000 rpm for 10 min, the supernatant was transferred to a new microtube and stored at -20°C until use.

**DNA extraction from strains stored in ethanol:** The DNA extraction was performed according to Montenegro et *al*. (2003) with minor modifications [12]. Briefly, 100 μl of bacterial suspension Mc Farland 1 was added in a microtube containing 500μl of absolute ethanol. The suspension was centrifuged at 12000×g for 5 minutes and the supernatant was discarded. The pellet was washed with 200 μl TE buffer and centrifuged at 12000×g for 10 minutes. The supernatant was discarded and 75 μl of 10% Chelex-100 was added to the microtube, mixed and heated at 95°C for 30 minutes. The microtube was then centrifuged at 13000 rpm for 10 minutes and the supernatant was transferred to a new microtube and stored at -20°C until use.

**16SrRNA PCR amplification:** All DNA extracted from ZN stained smear slide, GenoCard, FTA card and ethanol systems were first tested with 16SrRNA PCR amplification. PCR product was checked in 2% agarose gel electrophoresis.

**GenoType Mycobacterium CM:** The reverse line probe assay GenoType *Mycobacterium* CM was performed according to the manufacturer's instructions, using the reagents provided with the kits (Hain Lifescience, Nehren, Germany). The GenoType Mycobacterium CM protocol consists of PCR amplification, hybridization of the PCR products to the strips, detection of the hybridization bands, and interpretation of the results. The GenoType Mycobacterium CM permits the identification of the following mycobacterial species: *M*. *avium*, *M*. *chelonae*, *M*. *abscessus*, *M*. *fortuitum*, *M*. *gordonae*, *M*. *intracellulare*, *M*. *scrofulaceum*, *M*. *interjectum*, *M*. *kansasii*, *M*. *malmoense*, *M*. *marinum-M*. *ulcerans*, *M*. *peregrinum*, *M*. *tuberculosis* complex, and *M*. *xenopi*. GenoType *Mycobacterium* CM was performed to verify the correct identification of the 4 NTM used in the phase 1 from the DNA previously extracted from the 4 systems of storage.

#### Part 2: MTB DNA extraction

Six MTB strains (3 MDR and 3 susceptible) were used to validate the DNA extraction protocol from the 4 storage systems standardized in part 1. The same DNA extraction protocols were used as described in part 1 for each system. However, the GenoType MTBDR*plus* was performed for the detection of MTB with drug resistance to RIF and INH.

**GenoType MTBDRplus:** The GenoType MTBDR*plus* V2 was carried out according to the manufacturer's instructions (Hain Lifescience, Nehren, Germany). Briefly, for the amplification process, a total of 5 μl extracted DNA was added to 45μl of PCR mixture containing 10 μl of amplification Mix A and 35 μl of amplification Mix B provided with the kit. The amplification protocol consisted of 15 min of denaturing at 95°C, followed by 10 cycles comprising 30s at 95°C and 2min at 65°C, an additional 20 cycles comprising 25s at 95°C, 40s at 50°C, and 40s at 70°C, and a final extension at 70°C for 8 min. Hybridization, detection steps and interpretation of results were performed according to the manufacturer's instructions.

### Phase 2: Viability testing of MTB on different storage and transport system

Cell suspensions of 1mg/ml of MTB H37Rv reference susceptible strain (1x10^7^ CFU/ml) and, 10^−1^ and 10^−2^ dilutions were added to each transport system and tested for viability by inserting a paper disc of each cell suspension on an LJ medium or by inoculating a ZN-stained material scraped off from the microscopic slides on LJ medium and examining for growth for up to 42 days. Growth was quantitated as follows: “1+” if the number of colonies is between 50–100; “2+” if it’s between 100–200 colonies and “3+” if it’s more than 200 colonies or confluent colonies.

**In a first experiment**, to test the viability of bacteria spotted on slide, 100 μl of MTB H37Rv suspension (1 mg/ml) were spread on 2 slides and fixed by heating. After fixation, one of the slides was stained by ZN, and then the smear scraped off the slide using 25 μl of distilled water. The suspension was transferred to a microtube and decontaminated with sodium dodecyl (lauryl) sulphate-NaOH (SDS–NaOH), inoculated on LJ medium and incubated at 37°C for up to 42 days for colony counting. For the other slide, after fixation, the smear was directly removed without prior ZN staining, decontaminated, inoculated on LJ medium and incubated at 37°C for up to 42 days. For the FTA and GenoCard, 100 μl and 60 μl of MTB H37Rv suspension (1 mg/ml) were inoculated on each card, respectively, and left them dry at room temperature inside the biosafety cabinet. Viability testing was determined after different contact times with bacterial cells: directly after drying corresponding to time 0 (T0), after 15 min (T15), 30 min (T30), 1h (T1h) and 1 night (T 1 night). At each time point, 3 discs were punched, placed into 500 μl sterilized distilled water and mixed. The suspension was inoculated on LJ and incubated at 37°C for up to 42 days. The viability testing in ethanol was tested by inoculating 100 μl of MTB H37Rv suspension (1 mg/ml) in 500 μl of absolute ethanol. Directly at T0, 100 μl of the suspension was inoculated on LJ and incubated at 37°C for up to 42 days.

**In a second experiment,** according to the results observed in the first experiment and in order to ensure the safety of the GenoCard and FTA cards, the viability of the mycobacterial cells was tested by spotting a suspension of MTB H37Rv (1 mg/ml, 10^−1^ and 10^−2^) on GenoCard and FTA cards and inactivated by using different concentrations of ethanol. The same protocol as described in the first experiment was followed. After spotting the suspension at different dilutions, and after drying them, 100 μl of 70% ethanol was added to one card, 100 μl of 90% ethanol to a second card, 100 μl of absolute ethanol to a third card and the last card without ethanol was used as a control. For each test (GenoCard and FTA cards), after 1 hour and after 1 night of contact, 3 discs were punched and added to a microtube containing 500 μl distilled water and directly inoculated on LJ and incubated at 37°C for up to 42 days. Each experiment was performed twice.

### Phase 3: Field evaluation of the four different storage and supports

Each participating site selected 50 MTB clinical strains (25 MDR-TB and 25 susceptible). To confirm their resistance profile, they were subcultured on LJ medium for testing susceptibility to RIF and INH by the proportion method on LJ or in the MGIT960 system. The McFarland 1 suspension of MTB strains were spotted on the different storage supports to validate the DNA extraction protocol for the detection of MDR-TB using the GenoType MTBDR*plus* as described in phase I. All tests were performed blindly without knowing the resistance profile of the strains tested and results were compared to those obtained by the conventional DST method. Before starting, to ensure that the same study procedure was followed, the laboratory of microbiology from the University of Ghent prepared the standard operating protocol and distributed it to each site.

### Data analysis

The performance of the DNA extraction method from each storage support to detect RIF and INH resistance was assessed separately by calculating sensitivity and specificity with standard methods. Sensitivity was defined as the ability to detect true resistance, and specificity was defined as the ability to detect true susceptibility. For the mycobacterial viability testing, the standard deviation (± SD) was calculated using excel (average colonies numbers of 2 experiments).

## Results

### Phase 1: Standardization of DNA extraction from each storage and transport system

#### Part 1

**Fig 1:**
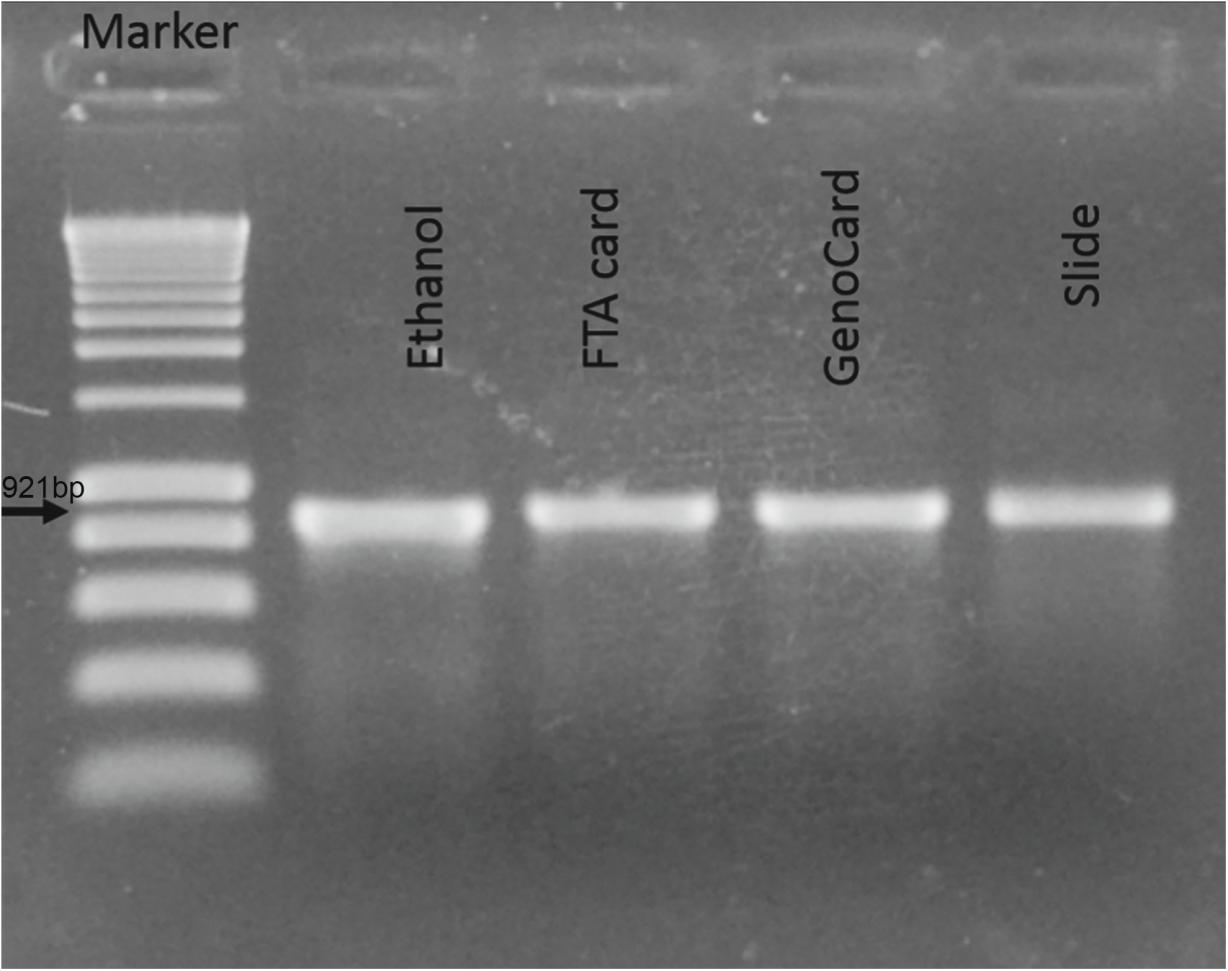
16SrRNA PCR results on 2% agarose gel. The appearance of 921 bp PCR amplicon indicates the positive results for MTB. M: marker, lane 1: from ethanol; lane 2: from FTA card; lane 4; from GenoCard; lane 4: from slide.

We first standardized and optimized the DNA extraction protocol from the different storage supports using NTM. For the DNA extraction from suspensions spotted on FTA cards, we initially found that 1 punched disc was not enough for PCR amplification. The 16SrRNA amplification product was negative in 2% agarose gel electrophoresis. However, DNA extraction from 4 discs by using the 10% Chelex-100 method showed a very good and an optimal PCR result (Fig 1). Similarly, DNA extraction from the mycobacterial cells spotted on GenoCard was standardized with 8 punched discs by using the 10% Chelex-100 method (Fig 1). Equally satisfactory results with or without 10% Chelex-100 were obtained for both cards. Nevertheless, the DNA extraction protocol using 10% Chelex-100 was selected for the field evaluation because it has the advantage of allowing having a sufficient amount of DNA (75μl) for subsequent additional tests, which is not possible using directly the punched discs in the PCR mix. The Chelex DNA extraction methods from ZN smear and from the strain kept in ethanol gave also satisfactory results (Fig 1). All NTM were correctly identified by GenoType Mycobacterium CM using the 4 storage and transport systems.

#### Part 2

Also, the six MTB strains were correctly identified as susceptible or resistant by the GenoType MTBDR*plus* from DNA extracted in the four systems of storage using also Chelex method.

### Phase 2: Viability testing of MTB on different storage and transport system

For the viability testing, LJ was inoculated from the different supports spotted with MTB H37Rv and results are presented in Table 1. After 42 days, no growth was observed on LJ slants which were inoculated from ZN stained smears and from the suspension kept in ethanol. However, visible growth on LJ was observed (1+ or 2+) from discs seeded with MTB H37Rv from the FTA card and GenoCard at the different contact times. In the second experiment, the average growth from FTA card and GenoCard after the addition of 70%, 90% and absolute ethanol is presented in Table 1. Visible growth was still observed in 70% ethanol at the two contact points and viability was not lost even at the lower bacterial concentration (10^−2^). Interestingly, no growth was observed using 90% ethanol or absolute ethanol.

**Table 1:** Mycobacterial viability testing.

**1** ^**st**^ **experiment: H37Rv**													
	Supports												
	**Slide**		**Ethanol**	**FTA card**					**GenoCard**				
	No ZN	ZN	T0	T0	T15	T30	T1h	T1 night	T0	T15	T30	T1h	T1 night
N° colonies on LJ	0	0	0	2+	1+	1+	1+	1+	2+	Cont.	2+	2+	2+
**2** ^**nd**^ **experiment: H37Rv + Ethanol**													
				Supports									
				**FTA card**					**GenoCard**				
N° colonies on LJ				T1h [± SD]			T1 night [± SD]		T1h [± SD]			T1 night [± SD]	
	Ethanol 70%		1 mg/ml	2+			34 ± 7.1		2+			71 ± 1.4	
			10^−1^	1+			1+		30 ± 1.4			5 ± 0.7	
			10^−2^	39 ± 4.2			3 ± 1.4		8 ± 7.1			0	
	Ethanol 90%		1 mg/ml	0			0		0			0	
			10^−1^	0			0		0			0	
			10^−2^	0			0		0			0	
	Absolute ethanol		1 mg/ml	0			0		0			0	
			10^−1^	0			0		0			0	
			10^−2^	0			0		0			0	

No ZN = no ZN staining, Cont. = contaminated, T0: time zero, T15: 15 min contact, T30: 30 min contact, T1h: 1 h contact, T1 night: 1 night contact. SD = Standard Deviation.

### Phase 3: Field evaluation of four different storage and supports

Results of the field evaluation are shown in Table 2. In total 200 strains were processed on four different supports at the four sites. From strains spotted on slides, a total of 195 were correctly identified by the GenoType MTBDR*plus*. DNA extraction was negative from one sample (site 2). The GenoType MTBDR*plus* assay was considered as invalid (no TUB signal) from 4 samples (site 3). For the GenoCard system, a total of 194 samples were correctly identified by GenoType MTBDR*plus*. Three samples gave invalid results (site 3) (no signal TUB) and the DNA extraction was negative from another three samples (site 2). For the FTA card all 200 strains were correctly identified by GenoType MTBDR*plus*. For strains stored in ethanol, 197 were correctly identified. DNA extraction was negative from 2 samples (site 2), and one invalid result was obtained by Genotype MTBDR*plus* (site 3). Table 3 shows the specificity and sensitivity obtained for each system. For both drugs an excellent agreement was obtained between the GenoType MTBDR*plus* and the reference DST method performed at each site. Three sites reported 100% sensitivity and 100% specificity. Site 4 obtained a sensitivity between 80% and 84% for both drugs and specificity between 88% and 100% for all supports. The overall sensitivity and specificity for all sites was of 95 to 100%.

**Table 2:** Susceptibility results of 50 *M*. *tuberculosis* strains obtained by Genotype MTBDR*plus* performed on the DNA extracted from different supports in each site compared to the LJ /MGIT960.

			Genotype MTBDRR*plus*							
			Slide		GenoCard		FTA card		Ethanol	
**Study sites**	**Drugs**	**LJ/MGIT960**	R	S	R	S	R	S	R	S
**Site 1**	RIF	R	25	0	25	0	25	0	25	0
		S	0	25	0	25	0	25	0	25
	INH	R	25	0	25	0	25	0	25	0
		S	0	25	0	25	0	25	0	25
**Site 2**	RIF	R	24	0	23	0	25	0	24	0
		S	0	25	0	24	0	25	0	24
	INH	R	24	0	23	0	25	0	24	0
		S	0	25	0	24	0	25	0	24
**Site 3**	RIF	R	22	0	22	0	25	0	25	0
		S	0	24	0	25	0	25	0	24
	INH	R	23	0	23	0	26	0	26	0
		S	0	23	0	24	0	24	0	23
**Site 4**	RIF	R	21	4	20	5	21	4	21	4
		S	0	25	0	25	0	25	1	24
	INH	R	20	5	20	5	21	4	20	5
		S	2	23	2	23	2	23	3	22

R = resistant, S = susceptible, RIF = Rifampicin, INH = Isoniazid

Site 2: slide n = 49 (1 sample excluded: DNA extraction negative); GenoCard n = 47 (3 samples excluded: DNA extraction negative); Ethanol n = 48 (2 samples excluded: DNA extraction negative)

Site 3: slide n = 46 (4 samples invalid: no signal TUB); Genocard n = 47 (3 samples invalid: no signal TUB); Ethanol n = 49 (1 invalid result).

**Table 3:** Specificity and sensitivity of the Genotype MTBDR*plus* performed on the DNA extracted from different support in each site compared to conventional method.

		Slide		GenoCard		FTA card		Ethanol	
Study sites	Drugs	Sens (%)	Spec (%)	Sens (%)	Spec (%)	Sens (%)	Spec (%)	Sens (%)	Spec (%)
**Site 1**	RIF	100	100	100	100	100	100	100	100
	INH	100	100	100	100	100	100	100	100
**Site 2**	RIF	100	100	100	100	100	100	100	100
	INH	100	100	100	100	100	100	100	100
**Site 3**	RIF	100	100	100	100	100	100	100	100
	INH	100	100	100	100	100	100	100	100
**Site 4**	RIF	84	100	80	100	84	100	84	96
	INH	80	92	80	92	84	92	80	88
**All sites**	RIF	96	100	95	100	96	100	96	99
	INH	95	98	95	98	96	98	95	97

Sens = sensitivity, Spec = specificity, RIF = Rifampicin, INH = Isoniazid

## Discussion

To our knowledge, this is the first multicenter field study that explores the use of four transport and storage systems simultaneously, demonstrating the feasibility of using DNA extracted from different supports for *M*. *tuberculosis* resistance testing with the GenoType MTBDR*plus* assay. We have to point out that the major limitation of this study is the use of *M*. *tuberculosis* strains and not of sputum samples. This was the purpose of this work, to demonstrate for the first time in a multicenter study evaluation the comparative performance of several transports systems before applying them to sputum samples. We have also addressed for the first time the safety issue of using these systems and set-up the best DNA extraction method to be used in the prospective field evaluation using sputum samples (ongoing study). Our study demonstrated that DNA extracted from these supports can be amplified successfully using the Chelex method and drug resistance correctly detected by the molecular test. Overall, high sensitivities and specificities in detecting RIF (96% to 100%) and INH (94% to 98%) resistance were obtained from DNA extracted from each storage system compared with the conventional DST method. Very few studies have evaluated these supports of storage and transport for molecular detection of drug resistance. Guio et al., (2006) amplified MTB IS*6110* region from sputum samples spotted on FTA cards and compared with the results of microscopy examination and found that the sensitivity and specificity of PCR using the FTA card system were higher than microscopy examination. Tortoli et al. (2008) evaluated the performance of the GenoCard using MTB strains and showed that all samples produced good quality amplification products and good 16SrDNA sequencing results [13]. Also, Miotto et al. (2008) evaluated the performance of the GenoCard for the identification of MDR-TB using the GenoType MTBDR*plus* and for molecular typing by Mycobacterial Interspersed Repetitive Units Variable Number Tandem Repeats (MIRU-VNTR) and found it a useful tool for transport and storage of samples for quick monitoring of drug-resistance [11]. In our study, few discordant results were found compared to the conventional method. Some strains were found resistant with the proportion method but susceptible by GenoType MTBDR*plus* and the opposite was also shown for a few strains. One possible explanation might be that phenotypic resistance was due to other gene mutations not incorporated into the strip of GenoType MTBDR*plus* [14–16]. We believe that the discordances could be attributed to the proportion method and not to the quality of the DNA extracted, since the results of the GenoType MTBDR*plus* from all supports were concordant. However, this study could be an innovative approach for the rapid detection of MDR-TB taking only 2 days for results. Moreover, resistance to second-line drugs such as fluoroquinolones and injectable drugs using the Genotype MTBDR*sl* should also be possible. It is important to point out that the bacilli may remain viable and confer an infection risk after spotting the cells on filter paper [17]. Transportation of MTB strains needs to be safe. Our data clearly showed that FTA and GenoCard did not inactivate MTB H37Rv strain. Even the FTA lysis buffer was shown to be not sufficiently effective in inactivating mycobacterial cells. Rajendram et al. (2006) also reported that the viability of cells retained on the FTA cards varied among broad groups of bacteria. For Gram negative species, no viable cells were retained even at high cell densities and for the most robust species such as spore-formers and acid-fast bacteria, complete inactivation was achieved only at low cell densities [18]. In our study, using 90% or absolute ethanol rendered bacteria seeded on filter paper cards completely non-viable. Therefore to ensure the safe handling of the GenoCard and FTA card, inactivation of infectious bacteria spotted onto the cards must be ensured by simply adding a drop of 90% ethanol or absolute ethanol. Without this treatment, FTA cards and GenoCard spotted with samples containing live MTB should be considered as potentially infectious and should be handled carefully. However, using smear microscopy, after staining the smears, no mycobacteria will remain viable and the smears become safe for handling, as reported earlier, which is in concordance with our results [19].

## Conclusion

Due to the simplicity and ease of transport, these support systems could represent an important economical and cost-effective alternative for subsequent molecular detection of drug resistance in TB. They have the potential for a widespread use in remote endemic areas where specimens have to be transported through long distances to the laboratory. They can be stored at room temperature until needed for molecular analysis; however, more work is needed to assess the DNA stability on these supports for a long period of time. The optimal DNA extraction method was explored and we recommend using 10% Chelex-100, which gave very good PCR and molecular results. Care must be taken to avoid cross-contamination when punching the discs between the cards. GenoCard has the advantage that no purification step is needed, the punched disc can be used directly for PCR. Besides, another great advantage is that the cards are easy to label and handle. Evaluation of the potential use of these four systems to detect RIF and INH resistance from direct sputum samples is under evaluation.
